# Personality Traits, Burnout, Workplace Bullying and Sleep Quality Among Midwives: A Pilot Study

**DOI:** 10.1002/nop2.70266

**Published:** 2025-08-21

**Authors:** Simona Dobešová Cakirpaloglu, Jiří Vévoda, Panajotis Cakirpaloglu, Šárka Vévodová, Peta Jane Greaves, Dorota Folwarczná

**Affiliations:** ^1^ Department of Humanities and Social Sciences Faculty of Health Science, Palacký University Olomouc Olomouc Czech Republic; ^2^ Department of Psychology Faculty of Arts, Palacký University Olomouc Olomouc Czech Republic; ^3^ Department of Nursing, Midwifery and Health Faculty of Health and Life Sciences, Northumbria University Newcastle upon Tyne UK

**Keywords:** burnout, midwifery, personality, psychosocial risks, sleep quality, workplace bullying

## Abstract

**Aim:**

The work of midwives is often mentally and physically exhausting. We sought to determine whether an association exists between personality traits, burnout syndrome, bullying in the workplace, and sleep quality. Additionally, we aimed to assess whether personality traits, dimensions of burnout, and sleep quality differ between a group of bullied and non‐bullied midwives.

**Design:**

A cross‐sectional, correlation study was conducted.

**Methods:**

The research sample consisted of 71 midwives, average age 36 years, average length of practice 14.23 years. Data were collected using a demographic questionnaire, Maslach Burnout Inventory (MBI), Negative Acts Questionnaire—revised (NAQ‐R), International Personality Item Pool: Big Five Markers—50 (IPIP‐BFM‐50) and Pittsburgh Sleep Quality Index (PSQI).

**Results:**

There was a significant relationship between personality traits, workplace bullying, the dimensions of burnout, and poor sleep quality. There were significant differences in extroversion, emotional stability, emotional exhaustion, and sleep quality between those midwives that experienced workplace bullying and those that did not. Victims of workplace bullying tended to be more neurotic and less extroverted than non‐victims.

**Conclusion:**

The study findings indicate that personality traits may function as both predictors and outcomes of workplace bullying.

There was no patient or public contribution.

## 1 Introduction

Occupational psychosocial risks arise from factors both inside and outside the workplace. The factors that trigger psychosocial risks in the workplace arecoping with demanding clients, long working hours, time pressure, poor communication and cooperation with colleagues, and high work demands (Seilerová 2019; Dollard et al. 2007). Psychosocial risks in the workplace are defined as aspects of the management of work and its organisation that can bring about psychological or physical harm, decrease work productivity, or increase absenteeism and sickness (Nielsen and Einarsen 2012; Goldberg et al. 2006). The European Agency for Safety and Health at Work has recognised burnout syndrome, work‐related stress, bullying, violence, and substance abuse as psychosocial risks (Nielsen and Einarsen 2012). The advanced qualification and competence requirements of midwives bring exposure to these risks.

## 2 Methodology

The aim of this study was to discover whether connections exist between personality traits, burnout syndrome, bullying in the workplace, and sleep quality, and whether there are differences in personal qualities and sleep quality in a group of bullied and non‐bullied midwives.

### 2.1 Materials and Methods

The work is a cross‐sectional study using a demographic questionnaire and four standardised questionnaires:

- Maslach Burnout Inventory (MBI),
- Negative Acts Questionnaire‐revised (NAQ‐R)
- International Personality Item Pool: Big Five Markers—50 (IPIP‐BFM‐50)
- Pittsburgh Sleep Quality Index (PSQI)

### 2.2 Ethics

The research was approved by the Ethics Committee of the “REDACTED”, and by Ethics Committees and managements of the respective hospitals. A written formal consent form for participation in this study was sent to all participants prior to undertaking the data collection. The consent form indicated that participation was voluntary and participants had the right to withdraw from the study at any time without any negative consequences for them. They were informed that we would only use the information for the purposes of the present study. The collected data and personal information have been treated confidentially and in accordance with the General Data Protection Regulations (GDPR). Presentation of the results will remain anonymous. The results will be presented at group level, and it will not be possible for participants to be identified in publications.

### 2.3 Sampling

As this is a pilot study, we did not use a sample representative of the Czech Republic as a whole. The investigation was conducted with the cooperation of the Czech Chamber of Midwives, which sent an email to its members explaining the project and providing the link to the online questionnaire. The survey itself was conducted from October–December 2021.

The sample was: midwives from the Czech Republic with a minimum of 1 year in practice. The final sample consisted of 71 respondents. Participation was voluntary and anonymity was ensured (Table 1).

**TABLE 1:** Characteristics of the participants.

Characteristics	Value
Number	71
Age (mean)	36 years
Years in practise (mean)	14.23 ± 12.81 (Min 1, Max 40)
Years with current employer (mean)	10.44 ± 10.62
Place of work	
Delivery room	28
Neonatal care	10
Gynaecology	11
Emergency gynaecology	8
High risk pregnancy	14
Shift pattern	
3 shift system—8 h morning, afternoon or night	83%
8 h morning shifts only	11%
Two alternating 12 h shifts	6%
Type of employment	
Private sector	3%
State Sector—University Hospitals	34%
Public Sector—Regional District or City Hospital	53%

### 2.4 Data Collection

The data collection was conducted electronically using Google Forms. Participation in the study was voluntary, and the anonymity was ensured. 71 questionnaires were returned. None of the questionnaires was excluded due to missing values.

### 2.5 Analysis

The results of the negative acts questionnaire were used to divide the sample into two groups according to the predicted risk that they would be bullied. Group N1 comprised employees at low risk of bullying (NAQ‐R score: 1, 2) and group N2 comprised employees at risk of bullying at the workplace (NAQ‐R score: 4, 5). The consequent sample included 51 individuals. The remaining 20 subjects, at intermediate risk of workplace bullying, were not included in the further analysis.

The statistics were calculated using the software MS Excel 2016 and IBM SPSS V19. An analysis of the results distribution confirmed normal data distribution; for this reason, a parametric statistical approach was selected. Relationships between parameters were measured by the Pearson coefficient of correlation. The MANOVA test was used to assess if there is a relationship between the likelihood of being bullied and the likelihood of not being bullied and the dependent variables (extroversion, agreeableness, conscientiousness, emotional stability, intellect, EE, DP, PA, sleep quality). All tests were performed at the 5% level of significance.

### 2.6 Psychological Tools Used for the Research Investigation

#### 2.6.1 International Personality Item Pool—Big Five Markers—50

The midwives' personality traits were examined by means of the IPIP‐BFM‐50 questionnaire. The Five Dimensions of the IPIP‐BFM‐50 are: extroversion, agreeableness, conscientiousness, emotional stability, intellect/imagination. It is composed of 50 items consisting of a stem and a five‐point Likert scale (1 = not present at all, 5 = present a great deal). The dimensions demonstrated a high level of reliability with an overall Cronbach's Alpha of 0.877 (Goldberg et al. 2006).

#### 2.6.2 Negative Acts Questionnaire—Revised

The Czech translation of the questionnaire NAQ‐R was used for evaluation of the occurrence of workplace bullying. Twenty‐two statements have a 5 point Likert scale rating how often the given negative act occurs (1 = never, 2 = occasionally, 3 = monthly, 4 = weekly, 5 = daily). The Czech translation of the NAQ‐R questionnaire has a high reliability and validity with an overall alpha of 0.915 (Cakirpaloglu et al. 2016; Notelaers and Einarsen 2013).

#### 2.6.3 Maslach Burnout Inventory

The MBI scale was used for evaluating the level of burnout syndrome (Maslach and Jackson 1981). There are twenty‐two statements with a seven‐point Likert scale for the intensity of feelings experienced by the respondent (0 = not at all, 7 = quite powerfully). The questionnaire is focused on three categories: emotional exhaustion (EE), personal accomplishment (PA) and depersonalization (DP). The respondents can be divided into two groups: those not at risk of burnout syndrome, and those at risk of burnout syndrome. The dividing values are established individually for each category. The Cronbach's alfa for the dimensions of the MBI inventory showed acceptable reliability (EE alfa 0.884, DE alfa 0.778, PA alfa 0.564, overall alfa 0.696).

#### 2.6.4 Pittsburgh Sleep Quality Index

Sleep quality was evaluated by means of the Pittsburgh index of sleep quality. This contains 10 self‐evaluating questions, with question no. 5 containing another 10 sub‐questions. There is a scale of 0 to 21 points where 0 means sleep quality without difficulties and 21 refers to serious problems in all aspects of sleep. The authors of the questionnaire recommend the score 5 as the dividing value, while Czech authors (Manková et al. 2021) recommend a score higher than 10. The international border of five points was used in this research analysis (Buysse et al. 1989). The overall reliability of this method is alpha 0.73 (Raniti et al. 2018).

#### 2.6.5 The Socio‐Demographic Questionnaire

The socio‐demographic questionnaire provides demographic data such as age, place of residence, year in practice, family status, and other variables.

## 3 Results

The Pearson correlation demonstrated a weaker significant negative relation between the level of emotional exhaustion and extroversion (*r* = −0.259*; *p* = 0.029), agreeableness (*r* = −0.260*; *p* = 0.029), and emotional stability (*r* = −0.464**; *p* = 0.000). Similarly, a significant negative correlation was determined between depersonalization and extroversion (*r* = −0.274*; *p* = 0.021), agreeableness (*r* = −0.339**; *p* = 0.004), and emotional stability (*r* = −0.327**; *p* = 0.005). A significant relationship was found once again between conscientiousness, intellect/imagination and level of depersonalization. A significant correlation was not found with the category of ‘personal accomplishment’, which is characterised by its reverse value, in particular in relation to the level of conscientiousness. A positive centrally strong correlation was determined between personal accomplishment and extroversion (*r* = 0.509**; *p* = 0.000), agreeableness (*r* = 0.447**; *p* = 0.000), emotional stability (*r* = 0.459**; *p* = 0.000), and also to intellect/imagination (*r* = 0.442**; *p* = 0.000). A positive correlation was found between (*r* = 0.327**; *p* = 0.005) and depersonalization (*r* = 0.285*; *p* = 0.016). No significant correlation was found between the level of personal accomplishment and sleep quality (Table 2).

**TABLE 2:** Pearson correlation coefficient for Maslach Burnout Inventory, personality traits and sleep quality.

Variable	Emotional exhaustion	Depersonalisation	Personal accomplishment	Extroversion	Agreeableness	Conscientiousness	Emotional stability	Intellect	Sleep quality
Emotional exhaustion	1	0.460**	−0.358**	−0.259*	−0.260*	−0.161	−0.464**	−0.090	0.327**
Depersonalisation		1	−0.385**	−0.274*	−0.339**	−0.086	−0.327**	−0.018	0.285*
Personal accomplishment			1	0.509**	0.447**	0.108	0.459**	0.442**	−0.164
Extroversion				1	0.371**	−0.036	0.467**	0.288*	−0.244*
Agreeableness					1	−0.134	0.196	0.331**	0.067
Conscientious‐ness						1	0.201	−0.161	−0.105
Emotional stability							1	0.107	−0.238*
Intellect/imagination								1	−0.099
Sleep quality									1

* Correlation is significant at the 0.05 level.

** Correlation is significant at the 0.001 level.

A significant negative correlation was found between emotional stability and workplace bullying (*r* = −0,303*; *p* = 0.010), and a positive correlation between workplace bullying and sleep quality (*r* = 0,328**; *p* = 0,005). A positive central strong correlation (*r* = 0,569**; *p* = 0,000) exists between emotional exhaustion and workplace bullying. It is therefore probable that people experiencing bullying in the workplace will experience emotional exhaustion to a greater extent, while in contrast, emotionally exhausted people will be more likely to succumb to bullying attacks. No correlation was found, however, between workplace bullying and depersonalization or personal work satisfaction.

The second aim of our study was to determine if bullied and non‐bullied midwives differ in personality traits and sleep quality. The actual ANOVA tests were carried out for each dependent variable. Pearson correlations were performed between all of the dependent variables. After the correlation test was conducted, the results showed that a meaningful pattern of correlations was observed among all of the dependent variables (all correlations were positive and in the moderate range; *p* < 0.01), suggesting the appropriateness of the MANOVA test. In addition, the Box's *M* value of 76.52 had an insignificant association, with a *p* value of 0.258. Therefore, for the purposes of MANOVA, the covariance matrices between the groups were assumed to be equal. The results (Table 3) indicate that a statistically significant difference exists between the group of midwives, who were was not subjected to bullying and the group which were subjected to bullying in the workplace. For the personal factors, a significant difference was determined between the group of midwives without demonstrable bullying, and the group of midwives with demonstrable bullying at the workplace in terms of extroversion and emotional stability. No differences were found between the other personal factors. A significant difference was also determined in the dimension of emotional exhaustion. Midwives, who were not subjected to bullying have a lower score than those who were bullied and sleep quality.

**TABLE 3:** MANOVA table.

Variables	Bullying binned	Mean	Std. deviation	*N*	F	Sig.	Partial Eta squared
Extroversion	Without bullying	34.28	8.466	39			
	Bullied	28.75	6.312	12	4.352	0.042	0.082
	Total	32.98	8.298	51			
Agreeableness	Without bullying	41.51	4.833	39			
	Bullied	41.92	4.122	12	0.068	0.795	0.001
	Total	41.61	4.639	51			
Conscientiousness	Without bullying	39.56	4.833	39			
	Bullied	39.25	6.312	12	0.033	0.856	0.001
	Total	39.49	5.151	51			
Emotional Stability	Without bullying	31.69	7.665	39			
	Bullied	25.58	4.833	12	6.741	0.012	0.121
	Total	30.25	7.526	51			
Intellect	Without bullying	35.82	6.034	39			
	Bullied	36.17	5.491	12	0.031	0.860	0.001
	Total	35.90	5.859	51			
Emotional exhaustion	Without bullying	16.46	9.955	39			
	Bullied	31.17	10.701	12	19.348	0.000	0.283
	Total	19.92	11.840	51			
Personal accomplishment	Without bullying	39.31	6.910	39			
	Bullied	37.42	7.489	12	0.661	0.420	0.013
	Total	38.86	7.020	51			
Depersonalisation	Without bullying	3.74	3.719	39			
	Bullied	6.08	4.420	12	3.325	0.074	0.064
	Total	4.29	3.976	51			
GLOBAL poor sleep quality: >=5 (CR research >=10)	Without bullying	6.79	3.381	39			
	Bullied	9.58	2.314	12	7.088	0.010	0.126
	Total	7.45	3.360	51			

## 4 Discussion

The first step in this study was to determine the relationship between burnout syndrome, personality traits, and sleep quality. The scores for “emotional exhaustion” and “depersonalization” correlated negatively with emotional stability, extroversion, and agreeableness. In contrast, they correlated positively with personal accomplishment and extroversion, agreeableness, emotional stability, and intellect/imagination. Our findings are consistent with previous studies, where a relationship was found between emotional exhaustion and neuroticism, emotional stability, and agreeableness (Parent‐Lamarche and Marchand 2018; Brown et al. 2019). These results indicate that individuals who are more introvert, emotionally vulnerable, or less affable can be under greater threat of emotional exhaustion. Whereas the data relating to the score for depersonalization correlated positively with personality traits, in particular extroversion, agreeableness, and emotional stability. Individuals with a high level of extroversion, agreeableness, or emotional stability are less prone to depersonalization. It is consequently possible that the higher the level of extroversion, agreeableness, emotional stability, or intellect/imagination the individual possesses, the more satisfied he or she will be in their work.

A significant correlation was also found between sleep quality and scores for burnout syndrome; specifically, emotional exhaustion and depersonalization. These results agree with earlier findings (Söderström et al. 2012; Vela‐Bueno et al. 2008), where a significant positive relation was determined between sleep quality and burnout. Health workers suffering from burnout had a higher global index of sleep quality, which is confirmed by, among other things, research in Turkey (Aydin Sayilan et al. 2020). On the other hand, the findings of a Vladič and Kren (2021), which showed a positive correlation between emotional exhaustion, depersonalization, sleep quality, and personal accomplishment, were not confirmed in our pilot research.

A significant correlation between workplace bullying and burnout was only found in the dimension of emotional exhaustion. These pilot results confirm previous findings indicating a high level of burnout specifically in the area of emotional exhaustion among mobbed employees (Srivastava and Dey 2020; Karsavuran and Kaya 2015).

Vévodová et al. (2020) argue that significant relationships exist between all elements of burnout syndrome and bullying at the workplace, with the strongest correlation being between emotional exhaustion and workplace bullying. It is therefore probable that people who have undergone bullying experience more emotional exhaustion; conversely, emotionally exhausted people more easily succumb to bullying attacks.

Our results draw attention to the lower emotional stability of victims of workplace bullying. This finding is confirmed by a study by Wilson and Nagy (2017) where a significant relationship between neuroticism and bullying in the workplace was also found; and by Nielsen and Knardahl (2015), who found a higher value of neuroticism among victims of bullying in the workplace.

Another issue of interest in our study was to explore the connection between bullying in the workplace and sleep quality. We found that victims of mobbing regularly suffer from worse sleep quality. Similar findings are mentioned in other studies (Magee et al. 2015; Sun et al. 2017). Research indicates that the victims of workplace bullying show worsening mental health, in particular decreased well‐being, increased anxiety, depression, and overall disorganisation of purposeful activity (Cakirpaloglu et al. 2016). This relationship can also work in reverse; that is, people who do not experience adequate sleep can feel tired or can be moody, and consequently, it can be difficult for them to manage their own emotions.

The final finding of our pilot study was the differences in the personality traits of bullied and non‐bullied midwives. Significant differences were shown in particular in terms of the level of emotional exhaustion, emotional stability, and extroversion and sleep quality. These findings are in accordance with the results of a Korean study (Kim et al. 2015), where a significant difference in the dimension of emotional exhaustion was found between bullied and non‐bullied employees, while in the study of Mitsopoulou and Giovazolias (2015) a significant relationship was found between neuroticism and reduced extroversion among bullied people.

### 4.1 Implications for Practice

Managers have to be conscious of the individual and organisational risk factors and apply the principles for prevention of bullying and burnout syndrome in the workplace. An environment should be created which encourages employees to express their concerns. Informing personnel about their rights and the creation of a positive atmosphere in health facilities greatly assists employees to be more open about difficulties and to create support groups. Managers have to continue monitoring, exposing, and solving workplace bullying, along with carrying out interventions in order to reduce the basic factors of burnout syndrome. Other measures are the provision of social support, access to coaching, improved communication, and the provision of opportunities to take part in innovation (REFS). Useful education and training should focus on safety at the workplace for both patients and staff, with the aim of limiting tragic events and errors in treatment, which in turn reduces stress and helps staff to better manage demanding situations.

### 4.2 Limitations

There are several limitations to the study that should be mentioned. First of all, the online data collection, despite all precautions, may bias the results in a certain way. At the same time, this is a cross‐sectional study where the changes in the variables over time cannot be observed. Cut‐off points are based on the US data as Czech population standards do not exist and, therefore, the Czech cut‐off points may be different. The results may be biased due to a low number of respondents included in the sample. Also, the sampling method used may not have been neutral. Midwives who are being bullied may have been keen to tell their stories which might lead to some skewing of the data.

## 5 Conclusion

In conclusion, this study has yielded several important findings. Workplace bullying was found to be related to burnout syndrome and, at the same time, neuroticism and extroversion can be viewed as predictors of the incidence of workplace bullying and burnout. These results should be seen as an introductory study into this broad topic that needs further attention.

## Author Contributions

**Simona Dobešová Cakirpaloglu:** data curation, conceptualization, methodology, writing – original draft preparation. **Jiří Vévoda:** methodology, software. **Panajotis Cakirpaloglu:** visualisation, investigation. **Šárka Vévodová:** supervision. **Peta Jane Greaves:** writing – reviewing and editing. **Dorota Folwarczná:** data collection, writing.

## Conflicts of Interest

The authors declare no conflicts of interest.

## Data Availability

The authors have nothing to report.
